# Free Fat Grafts in Endoscopic Skull Base Closure: A Clinical Outcome Analysis

**DOI:** 10.3390/jcm15082987

**Published:** 2026-04-15

**Authors:** Daniel Ilie Rotariu, Bogdan Florin Iliescu, Razvan Buga, Bogdan Ionut Dobrovat, Bogdan Costachescu

**Affiliations:** 1 Department of Neurosurgery, Grigore T. Popa University of Medicine and Pharmacy Iasi, 11 Universitatii St., 700115 Iasi, Romania; daniel-ilie.rotariu@umfiasi.ro (D.I.R.); bogdan.costachescu@umfiasi.ro (B.C.); 2 “Prof Dr N Oblu” Clinical Emergency Hospital Iasi, 2nd Ateneului St., 700309 Iasi, Romania; bugarazvan@yahoo.com (R.B.); bogdan-ionut.dobrovat@umfiasi.ro (B.I.D.)

**Keywords:** fat grafts, skull base, endoscopy, fat resorbtion

## Abstract

**Background**: Autologous fat grafts are widely used for skull base reconstruction following endoscopic endonasal surgery. However, uncertainty regarding their postoperative volumetric evolution may complicate the distinction between expected postoperative changes and residual or recurrent disease on imaging. **Methods**: We performed a retrospective volumetric imaging analysis of patients undergoing endoscopic endonasal skull base surgery with reconstruction using autologous fat and the 3F technique between 2013 and 2023. Fat graft volumes were measured on postoperative day 1 CT scans and on 3-, 9-, and 15-month postoperative MRI using standardized volumetric segmentation analysis (method described in detail in the main text). It should be noted that baseline measurements were derived from CT, whereas follow-up assessments were performed using MRI. Resorption rates were correlated with demographic, pathological, surgical, and postoperative variables. **Results**: Thirty-four patients met inclusion criteria. Mean initial fat graft volume was 3.01 ± 2.65 cm^3^. Overall, fat graft volume demonstrated a consistent temporal decline, with mean reductions of 56.8% at 3 months, 75.3% at 9 months, and 81.8% at 15 months. In subgroup analysis, differences in resorption were observed according to surgical approach, with higher resorption following transsellar compared with transtuberculum approaches (87.4% vs. 74.8% at 15 months, *p* = 0.042). These findings were closely related to graft compartment, although compartment and surgical approach showed substantial overlap in this cohort. No significant associations were detected between resorption rate and age, sex, comorbidities, postoperative CSF leak, extent of resection, or adjuvant radiotherapy. **Conclusions**: Autologous fat grafts used for skull base reconstruction demonstrate substantial early postoperative resorption followed by slower progressive volume loss. Recipient anatomical compartment was associated with differences in observed resorption patterns, although this relationship should be interpreted in the context of overlap with surgical approach and limited sample size. These findings may assist in improving interpretation of postoperative imaging by clarifying the expected temporal pattern of fat graft evolution after endoscopic skull base reconstruction.

## 1. Introduction

Endoscopic endonasal transsphenoidal surgery has become the preferred approach for selected sellar and midline skull base lesions, providing excellent tumor control with favorable visual and endocrinological outcomes [1,2]. Nevertheless, cerebrospinal fluid (CSF) leakage remains a significant source of postoperative morbidity, with reported rates varying depending on factors such as pathology type, defect size, intraoperative leak grade, and reconstruction technique, and reaching up to approximately 10% in higher-risk cases, carrying a risk of serious complications such as meningitis, pneumocephalus, and brain herniation [3,4].

To mitigate these risks, meticulous skull base reconstruction is essential, particularly in the presence of intraoperative or preoperative CSF leakage. Autologous fat grafting, typically harvested from the abdomen or thigh, represents one of the most commonly employed reconstruction techniques due to its biocompatibility, ease of harvest, low infection risk, and capacity to conform to complex osteodural defects [5,6,7]. When used as part of a multilayer reconstruction strategy, fat grafts effectively obliterate dead space and contribute to the establishment of a watertight seal [8,9]. Within this composite reconstruction, fat grafts play a distinct role in volumetric filling and support, which justifies their evaluation as an independent component of the repair.

Despite these advantages, the biological behavior of free fat grafts remains unpredictable. Fat graft resorption is a well-recognized phenomenon, and its variable rate complicates postoperative imaging interpretation. Previous studies have described qualitative changes in graft appearance over time; however, the literature remains limited by small sample sizes, heterogeneous methodologies, and a lack of standardized, longitudinal volumetric assessment. In particular, the temporal pattern and extent of fat graft resorption following endoscopic skull base reconstruction have not been consistently quantified. Progressive changes in graft volume and signal intensity on MRI may mimic tumor recurrence or scar tissue, posing diagnostic challenges [10,11]. A clearer understanding of these imaging patterns is therefore essential to distinguish normal postoperative evolution from pathological findings.

Given these considerations, the present study aims to quantitatively characterize the temporal radiological changes of autologous fat grafts following endoscopic endonasal skull base surgery using serial imaging, and to identify clinical and surgical factors associated with graft resorption. By focusing specifically on the imaging behavior of the fat graft component, this work seeks to provide novel data that may improve the interpretation of postoperative MRI and support more accurate differentiation between expected healing changes and disease-related findings.

## 2. Materials and Methods

### 2.1. Study Design and Patient Selection

We conducted a retrospective analysis of consecutive patients undergoing endoscopic endonasal skull base surgery at the Neurosurgical Department of “Prof. Dr. N. Oblu” Clinical Emergency Hospital, Iași, Romania, between January 2013 and December 2023.

Inclusion criteria were: (1) adult patients (≥18 years), (2) skull base reconstruction performed using the 3F (fat–flap-fast mobilisation) technique, and (3) availability of complete postoperative imaging datasets (baseline CT and follow-up MRI) suitable for volumetric analysis.

Exclusion criteria were: (1) use of reconstruction techniques other than the 3F method, (2) incomplete or missing DICOM imaging data, (3) follow-up imaging not performed within predefined acceptable time windows, and (4) loss to follow-up or death prior to completion of the imaging surveillance period.

For the purpose of this study, a “true 3F reconstruction” was defined as a multilayer closure consisting of autologous fat graft for cavity filling, covered by a vascularized nasoseptal flap with the edges secured with fibrin glue and the 3rd F coming from (FLASH) meaning fast mobilization after the surgery. Cases in which one or more of these components were absent or replaced by alternative materials (e.g., synthetic grafts or non-vascularized flaps) were excluded.

**Figure 1:**
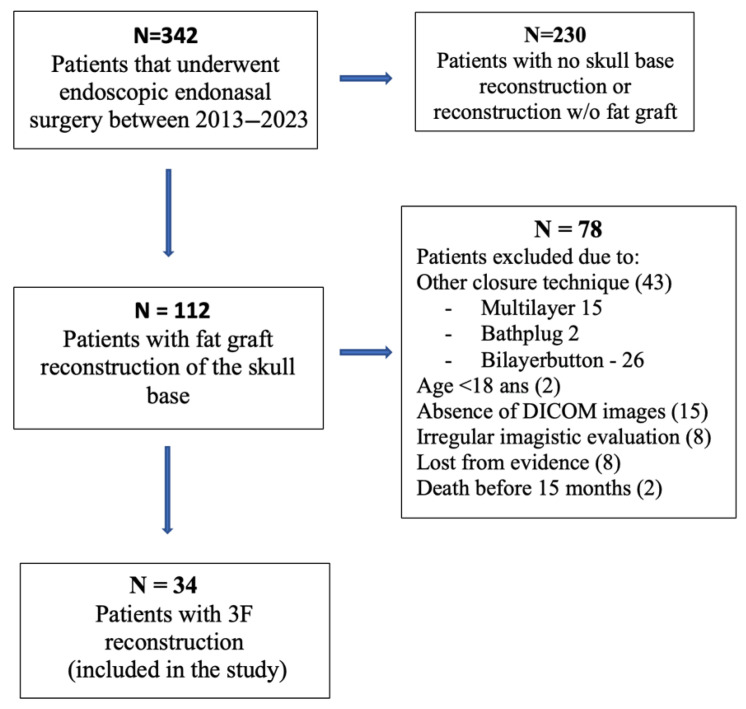
Flowchart illustrating patient selection. Of 342 endoscopic endonasal skull base procedures performed between 2013 and 2023, 112 involved reconstruction using autologous fat grafts. After applying predefined inclusion and exclusion criteria—including reconstruction technique, availability of complete imaging datasets, and adequate follow-up—34 patients were included in the final volumetric analysis.

Follow-up MRI examinations were scheduled at approximately 3, 9, and 15 months postoperatively. A deviation of ±6 weeks from each time point was considered acceptable. Patients with imaging performed outside these intervals were excluded, and the number of exclusions related to timing is illustrated in Figure 1.

### 2.2. Surgical Technique and Postoperative Management

All procedures were performed by the same surgical team using standardized transsellar or extended transtuberculum approaches, depending on lesion location and extent.

Autologous fat was harvested from the periumbilical region under sterile conditions, briefly soaked in gentamicin solution, and used to fill the surgical cavity and seal the osteodural defect. Reconstruction was completed using the 3F technique, consisting of (1) intradural fat graft placement for dead space obliteration, (2) vascularized nasoseptal flap secured in place with fibrin glue and maintained in position with a foley catheter.

Postoperatively, patients were mobilized early on day 1, after completion of the routine CT imaging performed to asses the degree of resection, the integrity of the skull base reconstruction and the presence of possible complication at the surgical site.

### 2.3. Imaging Protocol and Volumetric Analysis

Initial fat graft volume was calculated using postoperative day 1 CT scans, which served as the baseline reference. Follow-up volumetric measurements were obtained from MRI examinations performed at approximately 3, 9, and 15 months postoperatively using T1-weighted volumetric sequences.

Volumetric analysis was performed using Horos software (LGPL-3.0) through manual slice-by-slice segmentation of the fat graft. Graft boundaries were defined based on characteristic signal intensity and anatomical location, with correlation to fat-suppressed sequences to improve delineation from surrounding tissues. In cases with poorly defined margins, consensus between observers was reached through joint review.

Because baseline measurements were derived from CT and follow-up measurements from MRI, special care was taken to ensure comparability. Fat graft identification on CT relied on its characteristic low attenuation, while MRI identification was based on T1 hyperintensity and suppression on fat-saturated sequences. Although no formal cross-modality calibration was performed, consistent anatomical landmarks and standardized segmentation criteria were used across all time points.

All measurements were independently performed by three observers (two neurosurgeons and one neuroradiologist). Interobserver agreement was assessed using the intraclass correlation coefficient (ICC), and mean values were used for subsequent analysis.

Postoperative imaging protocols remained broadly consistent throughout the study period, with no major changes in acquisition parameters that would significantly affect volumetric assessment.

The primary endpoint of the study was the percentage change in fat graft volume over time relative to baseline.

Additionally, graft location was classified a priori into: (1) intrasellar (confined below the diaphragma sellae), and (2) suprasellar/subarachnoid (extending above the diaphragma), with additional notation of contact with the third ventricle when present.

### 2.4. Statistical Analysis

Continuous variables are presented as means with ranges, and categorical variables as frequencies and percentages.

Given the longitudinal nature of the data, repeated volumetric measurements within the same patient were analyzed using appropriate methods for correlated observations, including repeated-measures analysis. Linear regression was used to explore associations between continuous predictors and graft resorption rates.

Group comparisons for continuous outcomes (e.g., volume change between anatomical groups) were performed using Student’s *t*-test or non-parametric equivalents where appropriate.

Interobserver reliability was assessed using the intraclass correlation coefficient (ICC).

All tests were two-tailed, and statistical significance was defined as *p* < 0.05. Analyses were performed using IBM SPSS Statistics version 21 (IBM Corp., Armonk, NY, USA).

Given the limited sample size (*n* = 34), all analyses were considered exploratory, and no formal adjustment for multiple comparisons was performed.

### 2.5. Ethical Considerations

This study was conducted in accordance with the Declaration of Helsinki and approved by the local institutional ethics committee. Due to the retrospective design and use of anonymized imaging data, the requirement for informed consent was waived. This statement has been applied consistently throughout the manuscript.

## 3. Results

### 3.1. Patient Population and Baseline Characteristics

From a total of 342 endoscopic endonasal skull base procedures, autologous fat grafts were used in 112 cases. After applying the predefined inclusion and exclusion criteria, 34 patients were included in the final analysis (Figure 1).

The cohort included 9 males (26.5%) and 25 females (73.5%), with a mean age of 46.7 years (range 24–66).

Baseline demographic and clinical characteristics are summarized in Table 1 Comorbidities included diabetes mellitus in 3 patients (8.8%), arterial hypertension in 7 (20.6%), obesity (BMI > 30) in 13 (38.2%), and anticoagulant therapy in 3 (8.8%). Preoperative hydrocephalus was present in 5 patients (14.7%).

Surgical indications included 18 pituitary adenomas (52.9%), 6 craniopharyngiomas (17.6%), and 10 meningiomas (29.4%), with consistent reporting across text and tables.

### 3.2. Surgical Outcomes and Postoperative Course

Gross total resection was achieved in 21 patients (61.8%), near-total resection in 6 (17.6%), subtotal resection in 6 (17.6%), and partial resection in 1 case (2.9%).

Postoperative diabetes insipidus occurred in 16 patients (47.1%), including 11 transient and 5 permanent cases.

Postoperative CSF leakage occurred in 3 patients (8.8%), of whom one required surgical reintervention and two were managed with lumbar drainage.

Other complications included 3 cases of bacterial meningitis, all successfully treated with antibiotics, and one case of anterior epistaxis.

Visual outcomes at early follow-up showed improvement in 30 patients (88.2%), unchanged vision in 3 (8.8%), and deterioration in 1 patient (2.9%).

During follow-up, 7 patients (20.6%) received adjuvant radiotherapy (3 craniopharyngiomas and 4 pituitary adenomas).

### 3.3. Volumetric Analysis of Fat Graft Resorption

Baseline fat graft volume measured on postoperative day 1 CT was 3.01 ± 2.65 cm^3^. Mean graft volumes at follow-up were: 1.31 ± 1.33 cm^3^ at 3 months, 0.74 ± 0.84 cm^3^ at 9 months, 0.49 ± 0.69 cm^3^ at 15 months

These findings are summarized in Table 2.

**Figure 2:**
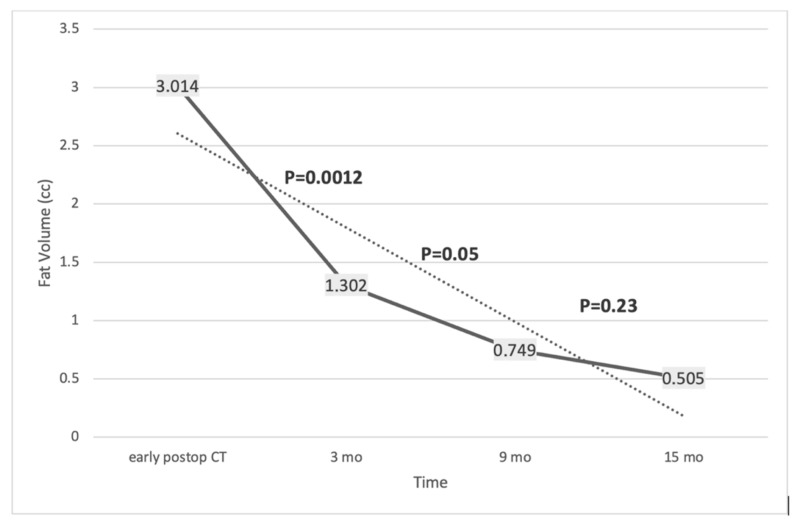
Temporal evolution of mean fat graft volume following reconstruction. Mean graft volume decreased progressively from baseline (postoperative day 1 CT) to 3, 9, and 15 months (MRI). Error bars represent standard deviation. Values correspond to descriptive cohort means; no inferential statistical testing is displayed in this figure (solid line displays the trend of the actual volumes, while the dashed line is the linear trend of the values).

**Figure 3:**
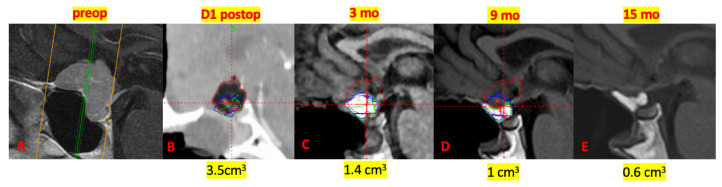
Representative case of fat graft evolution following reconstruction for a tuberculum sellae meningioma. (**A**) Intraoperative view after tumor resection and reconstruction. (**B**) Postoperative day 1 CT demonstrating fat graft in situ (volume: 3.5 cm^3^). (**C**–**E**) Follow-up MRI showing progressive reduction in graft volume at 3 months (1.4 cm^3^), 9 months (1.0 cm^3^), and 15 months (0.6 cm^3^) (red delineates the fat volume in day 1, blue at 3 months, and green at 9 months).

**Figure 4:**
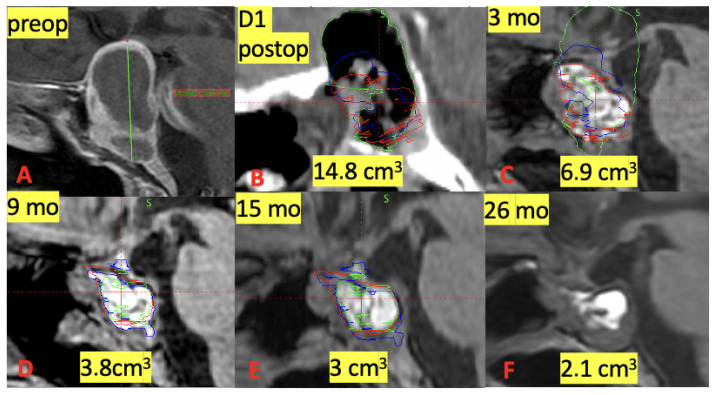
Representative case illustrating volumetric evolution of a fat graft following reconstruction for pituitary adenoma. (**A**) Preoperative MRI demonstrating sellar lesion. (**B**) Postoperative day 1 CT showing fat graft placement (volume: 14.8 cm^3^). (**C**–**E**) Progressive reduction in graft volume at 3, 9, and 15 months. (**F**) Late follow-up demonstrating residual graft volume at 26 months (green delineates fat volume in day 1 postop, blue at 3 months, and red at 9 months).

The greatest reduction in graft volume occurred within the first 3 months, with a mean decrease of 56.8% relative to baseline. Subsequent reductions were 42.8% between 3 and 9 months and 34.1% between 9 and 15 months, indicating a progressive but decelerating resorption pattern (Figure 2, Figure 3 and Figure 4).

At 15 months, the overall mean reduction in graft volume corresponded to 81.8% of the initial volume.

Qualitative imaging assessment demonstrated a progressive decrease in graft thickness with adaptation to the skull base defect, resulting in a more flattened configuration over time.

### 3.4. Factors Associated with Fat Graft Resorption

Results of subgroup analyses and statistical testing are presented in Table 3.

No statistically significant associations were identified between graft resorption rate and age, sex, comorbidities, postoperative CSF leak, extent of resection, or adjuvant radiotherapy (all *p* > 0.05).

Exploratory analysis using linear regression did not demonstrate significant associations between these variables and percentage volume reduction; corresponding regression coefficients were small and not statistically significant.

#### 3.4.1. Pathology

At 3 months, higher resorption rates were observed in pituitary adenomas compared with meningiomas and craniopharyngiomas (68.8% vs. 47.8% vs. 49.2%; *p* = 0.020).

**Figure 5:**
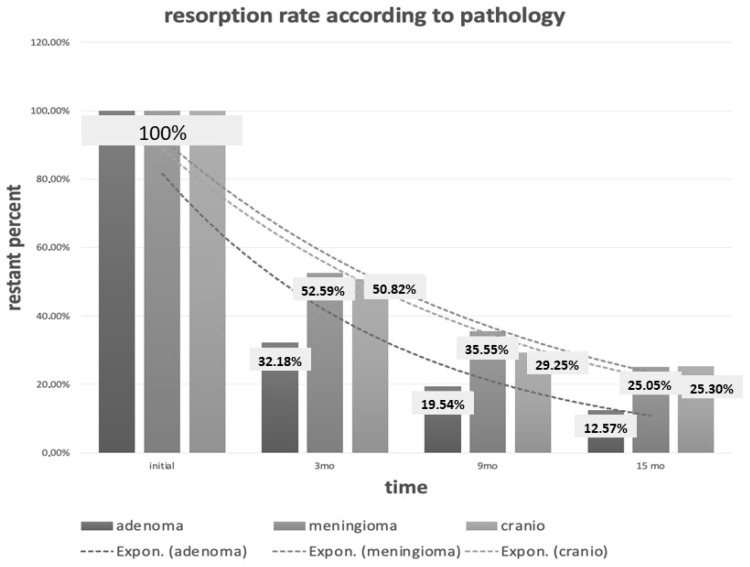
Fat graft resorption according to underlying pathology. Mean percentage volume reduction is shown for pituitary adenomas, meningiomas, and craniopharyngiomas at different time points. Differences between groups at individual time points were assessed using exploratory between-group comparisons (one-way ANOVA). Early differences were observed but were not consistently maintained across all follow-up intervals.

At 15 months, differences between groups persisted numerically but were no longer statistically significant (88.6% vs. 72.7% vs. 74.7%; *p* = 0.084) (Figure 5).

#### 3.4.2. Surgical Approach and Graft Compartment

**Figure 6:**
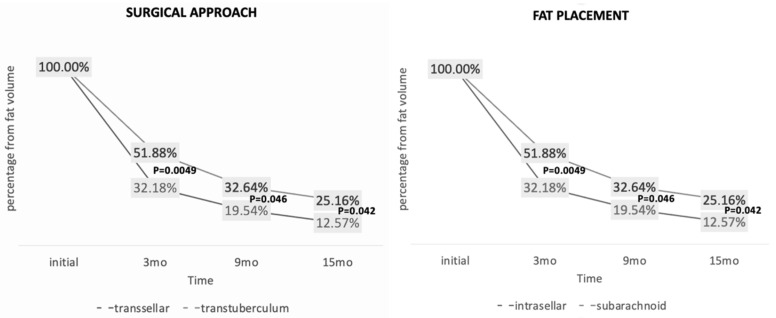
Fat graft resorption according to surgical approach and anatomical compartment. Mean percentage volume reduction is shown for transsellar versus transtuberculum approaches and for intrasellar versus subarachnoid graft placement. P-values represent exploratory between-group comparisons at each time point and should be interpreted cautiously, as these analyses are cross-sectional and do not account for repeated measurements over time. Substantial overlap between surgical approach and graft compartment is present in this cohort.

Fat graft resorption differed significantly according to surgical approach: transsellar approach: higher resorption rates vs. transtuberculum approach: lower resorption rates (Figure 6).

This difference was significant at all time points:

- 3 months: 67.8% vs. 48.2% (*p* = 0.004)
- 9 months: 80.5% vs. 66.5% (*p* = 0.040)
- 15 months: 87.4% vs. 74.8% (*p* = 0.042)

A similar pattern was observed for graft location, with higher resorption in intrasellar compared with subarachnoid placement (87.4% vs. 74.8%; *p* = 0.042).

However, these variables showed substantial overlap, as surgical approach strongly correlated with graft compartment, and therefore should not be interpreted as independent predictors.

#### 3.4.3. Contact with the Third Ventricle

In a small subgroup of 5 patients with graft contact with the third ventricle, no statistically significant association with resorption rate was identified (*p* = 0.069). Given the limited sample size, this finding should be considered exploratory.

### 3.5. Summary of Key Findings

Overall, fat graft resorption followed a consistent temporal pattern characterized by rapid early volume loss followed by slower progressive reduction. Significant differences were observed according to surgical approach and graft location, whereas no statistically significant associations were detected for demographic or clinical variables in this cohort.

## 4. Discussion

Autologous fat grafting is a well-established and widely adopted technique for skull base reconstruction following endoscopic endonasal surgery, owing to its biocompatibility, ease of harvest, and effectiveness in obliterating dead space and preventing cerebrospinal fluid (CSF) leakage [12,13,14,15]. In the present retrospective study, we provide a quantitative description of the postoperative volumetric evolution of fat grafts and explore potential factors associated with their resorption, while acknowledging the exploratory nature of the analysis and the limited sample size.

At the cohort level, fat graft resorption followed a consistent overall temporal trend, characterized by rapid early volume loss followed by a slower, progressive reduction over time. More than half of the initial graft volume was resorbed within the first three postoperative months, with total resorption reaching approximately 82% at 15 months. However, this overall pattern coexisted with marked interindividual variability, as reflected by the wide dispersion of values at each time point. Some patients demonstrated relatively high residual volumes at late follow-up, whereas others exhibited near-complete early resorption.

These findings are broadly consistent with previous radiological studies reporting progressive fat graft resorption over time [16,17,18]. However, direct comparison should be interpreted with caution, as prior reports include heterogeneous patient populations, encompassing not only sellar reconstruction but also extended skull base and lateral skull base procedures, where recipient site characteristics and surgical techniques may differ substantially. Isolated reports have documented residual fat grafts within the sellar region even many years after surgery, supporting the concept that a small proportion of adipose tissue may persist long term [19].

The biological behavior of free fat grafts is complex and likely multifactorial. Mechanisms such as early ischemic changes, macrophage-mediated resorption, and variable degrees of tissue integration and neovascularization have been described in the broader fat grafting literature. However, these processes were not directly assessed in the present study and should therefore be considered as general explanatory frameworks rather than findings derived from our data.

In this context, the observed association between graft resorption and anatomical compartment is of particular interest. Fat grafts placed predominantly in the intrasellar compartment exhibited higher resorption rates compared with those placed in the subarachnoid space. While this finding may suggest that local microenvironmental factors influence graft survival, including differences in vascular supply or tissue contact, such interpretations remain hypothetical and cannot be confirmed by the present data.

Importantly, surgical approach and graft compartment were closely related variables in this cohort, with substantial overlap between transsellar approaches and intrasellar placement, and between transtuberculum approaches and subarachnoid placement. This limits the ability to interpret these factors as independent predictors and should be taken into account when considering these results.

Conversely, cases of rapid or near-complete early resorption may reflect unfavorable local conditions. Potential contributing factors—including mechanical compression, CSF dynamics, graft handling techniques, or packing density—were not specifically measured in this study and are therefore proposed only as possible explanations. Similarly, local inflammatory responses or fat necrosis may play a role, although these mechanisms were not directly evaluated.

From a radiological standpoint, the interpretation of fat graft evolution remains challenging. Progressive changes in graft volume and signal characteristics on MRI may complicate differentiation between residual graft, postoperative scarring, and tumor recurrence [16,19]. This issue is further compounded by the use of different imaging modalities across time points in the present study, with CT used for baseline assessment and MRI for follow-up, which may introduce additional variability in volumetric estimation. Although fat-suppression sequences improve graft delineation, very small residual grafts may become difficult to distinguish from surrounding postoperative changes, potentially leading to overestimation of resorption.

In this cohort, postoperative complications related to reconstruction were infrequent, and the rate of CSF leakage was low. While these findings are reassuring, the study was not specifically designed to assess reconstructive durability or clinical efficacy, and no formal endpoint addressing structural integrity was defined.

Similarly, although no association was identified between initial graft volume and complication rates, the study did not include a formal analysis of “overpacking,” and this observation should therefore be interpreted with caution.

The present findings primarily contribute to the understanding of the radiological behavior of autologous fat grafts over time, rather than establishing their safety or comparative effectiveness within reconstruction strategies.

This study has several limitations. In addition to its retrospective design, relatively small sample size, and limited follow-up duration, several methodological constraints should be acknowledged: (1) the use of CT at baseline and MRI at follow-up, which may affect volumetric comparability; (2) the absence of formally reported interobserver agreement despite multiple raters; (3) the non-independence of surgical approach and graft compartment; and (4) the limited statistical modeling of repeated measurements in a small cohort. These factors may influence both the precision and generalizability of the results.

Future studies should aim to address these limitations through prospective longitudinal designs with standardized imaging modalities across all time points, predefined segmentation protocols, and formal assessment of interobserver reliability. In addition, larger cohorts would allow more robust modeling of repeated volumetric measurements and better evaluation of potential predictive factors.

## 5. Conclusions

Autologous fat grafts used in endoscopic endonasal skull base reconstruction demonstrate a consistent pattern of early rapid resorption followed by slower long-term volume reduction. An association between graft resorption and anatomical placement was observed, although this finding should be interpreted cautiously given the limited sample size and variable overlap with surgical approach.

These results support the role of fat grafts as a component of multilayer reconstruction and emphasize that expected postoperative resorption should be considered when interpreting follow-up imaging, particularly in distinguishing normal healing from residual or recurrent disease.

## Figures and Tables

**Table 1:** Biometric and medical data of the patient cohort included in the study.

Variable	Value
Number of patients	34
Age, mean (range)	46.7 (24–66)
Sex	
–Male	9 (26.5%)
–Female	25 (73.5%)
Pathology	
–Pituitary adenoma	18 (52.9%)
–Craniopharyngioma	6 (17.6%)
–Meningioma	10 (29.4%)
Comorbidities	
–Diabetes mellitus	3 (8.8%)
–Arterial hypertension	7 (20.6%)
–Obesity (BMI > 30)	13 (38.2%)
–Anticoagulant therapy	3 (8.8%)
Preoperative hydrocephalus	5 (14.7%)
Surgical approach	
–Transsellar	19 (55.9%)
–Transtuberculum	15 (44.1%)
Graft compartment	
–Intrasellar	19 (55.9%)
–Subarachnoid	15 (44.1%)

**Table 2:** Fat graft volume evolution in time.

Time Point	Mean Volume (cm^3^) ± SD%	Reduction from Baseline
Postop Day 1 (CT)	3.01 ± 2.65	—
3 months (MRI)	1.31 ± 1.33	56.8%
9 months (MRI)	0.74 ± 0.84	75.4%
15 months (MRI)	0.49 ± 0.69	81.8%

**Table 3:** Summary of statistical analysis findings.

*Variable*	*Groups*	*Mean Resorption (%)*	*p-Value*
*Sex*	Male	80.3%	0.62
	Female	83.8%	
*Pathology*	Pituitary adenoma	88.6%	0.084
	Craniopharyngioma	74.7%	
	Meningioma	72.7%	
*Surgical approach*	Transsellar	87.4%	0.042
	Transtuberculum	74.8%	
*Graft compartment*	Intrasellar	87.4%	0.042
	Subarachnoid	74.8%	
*Comorbidities*	Yes	81.5%	0.60
	No	79.2%	
*Postoperative CSF leak*	Yes	86.0%	0.30
	No	83.0%	
*Extent of resection*	GTR	85.0%	0.65
	NTR	74.0%	
	STR	82.3%	
	PR	97.0%	

## Data Availability

The original contributions presented in this study are included in the article. Further inquiries can be directed to the corresponding author.

## Abbreviations

The following abbreviations are used in this manuscript:

CSF	cerebrospinal fluid
GTR	gross total resection
NTR	near total resection
STR	subtotal resection
PR	partial resection
